# Comparison of surgical outcomes between lateral and posterior methods for retroperitoneoscopic adrenalectomy: insights from a single center’s experience

**DOI:** 10.3389/fonc.2026.1683516

**Published:** 2026-02-10

**Authors:** Hongliang Que, Zhongru Fan, Junpeng Deng, Quan Li, Tengyue Zeng, Qijie Zhang, Ke Wang, Jianjun Xie

**Affiliations:** Department of Urology, The Affiliated Suzhou Hospital of Nanjing Medical University, Suzhou Municipal Hospital, Gusu School, Nanjing Medical University, Suzhou, China

**Keywords:** adrenal gland, adrenalectomy, minimal invasive, propensity score match, retroperitoneoscopy

## Abstract

**Purpose:**

Lateral retroperitoneoscopic adrenalectomy (LRA) and posterior retroperitoneoscopic adrenalectomy (PRA) are both minimally invasive approaches for adrenal gland resection without entering the peritoneal cavity. Direct comparisons of their perioperative outcomes remain limited. This study evaluates the safety and efficacy of LRA versus PRA for adrenal tumor management.

**Methods:**

We retrospectively analyzed data from 185 patients undergoing LRA (n=95) or PRA (n=90) at a single center between January 2018 and May 2023. The cohort had a mean age of 53.8 ± 13.0 years, BMI of 24.8 ± 3.27 kg/m², and 50.3% (n=93) were male. Median tumor diameter was 2.3 cm (range: 1.0–6.5 cm). Perioperative parameters, complications, and outcomes were compared between groups. Propensity score matching (PSM) method was used to balance the potential confounding variables.

**Results:**

PRA demonstrated significantly shorter operative time (56.2 ± 13.7 vs. 79.0 ± 22.8 minutes; *p* < 0.001) and postoperative hospitalization (4.48 ± 1.50 vs. 5.91 ± 1.79 days; *p* < 0.001) compared to LRA. Hemoglobin change (1.32 ± 0.50 vs. 1.20 ± 0.28 g/dL; *p* = 0.060) and complication rates were comparable between groups. No cases required conversion to open surgery or resulted in mortality. PSM analysis validated the stability of these results. Multivariate logistic regression analysis indicated that BMI, being male, and the LRA approach were associated with operative time exceeding 60 minutes. Prolonged operative time and LRA were associated with extended hospital stays.

**Conclusions:**

Both LRA and PRA are safe and effective for adrenal tumor resection. Meanwhile, PRA may offer superior efficiency, with reduced operative duration and hospitalization, suggesting its potential as the preferred approach in select patients.

## 1 Introduction

Since Michel Gagner introduced the first successful laparoscopic adrenalectomy in 1992 (1), laparoscopic transperitoneal adrenalectomy (LTA) has become the gold standard for most adrenal disorders. It offers fewer complications, less pain, shorter hospital stays, and quicker recovery than open surgery (2, 3). To minimize intraabdominal invasion, retroperitoneoscopic adrenalectomy (RA) was developed, providing direct access to the retroperitoneum and adrenal gland without mobilizing intra-abdominal organs (4). Despite a limited surgical field and steeper learning curve compared to LTA, RA offers shorter operative times, less blood loss, reduced postoperative pain, faster recovery, and comparable complications for adrenal tumors (5–7).

Lateral (LRA) and posterior retroperitoneoscopic adrenalectomies (PRA) are both minimally invasive techniques for adrenal gland removal without entering the peritoneal cavity which differ mainly in the surgical methods and patient positioning (8, 9). To date, few studies have compared the clinical outcomes of LRA and PRA (10, 11). Therefore, this study aims to compare LRA and PRA in patients with adrenal tumors in a single Chinese medical center, focusing on surgical feasibility, efficiency, safety, and postoperative recovery.

## 2 Methods

### 2.1 Study population and perioperative management

The clinical data of 185 patients who underwent LRA or PRA at the Affiliated Suzhou Hospital of Nanjing Medical University from January 2018 to May 2023 were retrospectively reviewed. Four experienced urologists performed all operations and used both LRA and PRA techniques. This study was conducted in accordance with the Declaration of Helsinki and approved by the Institutional Ethical Review Board of Affiliated Suzhou Hospital of Nanjing Medical University (approval number: KL901440 and approval date: 10 January 2024).

Patient demographics were collected, including age, sex, body mass index (BMI), medical history of hypertension and diabetes mellitus, as well as American Society of Anesthesiologist (ASA) physical status classification. Standard imaging techniques determined tumor size, and endocrine investigations identified preoperative tumor pathology. Postoperative hemoglobin was tested in 24 hours to evaluate the blood loss, as well as electrolytes. The severity of surgery complications was evaluated by the Clavien–Dindo classification (12). Typically, a drainage tube can be removed if the postoperative fluid output is less than 20 mL (if one has been placed). Patients can usually be discharged 1–2 days after removal of the drainage tube, provided their postoperative cortisol levels are within normal range. Perioperative variables, including operative time, hemoglobin changes, surgical complications, and postoperative stay length, were recorded.

### 2.2 Operation techniques

#### 2.2.1 LRA

**Figure 1:**
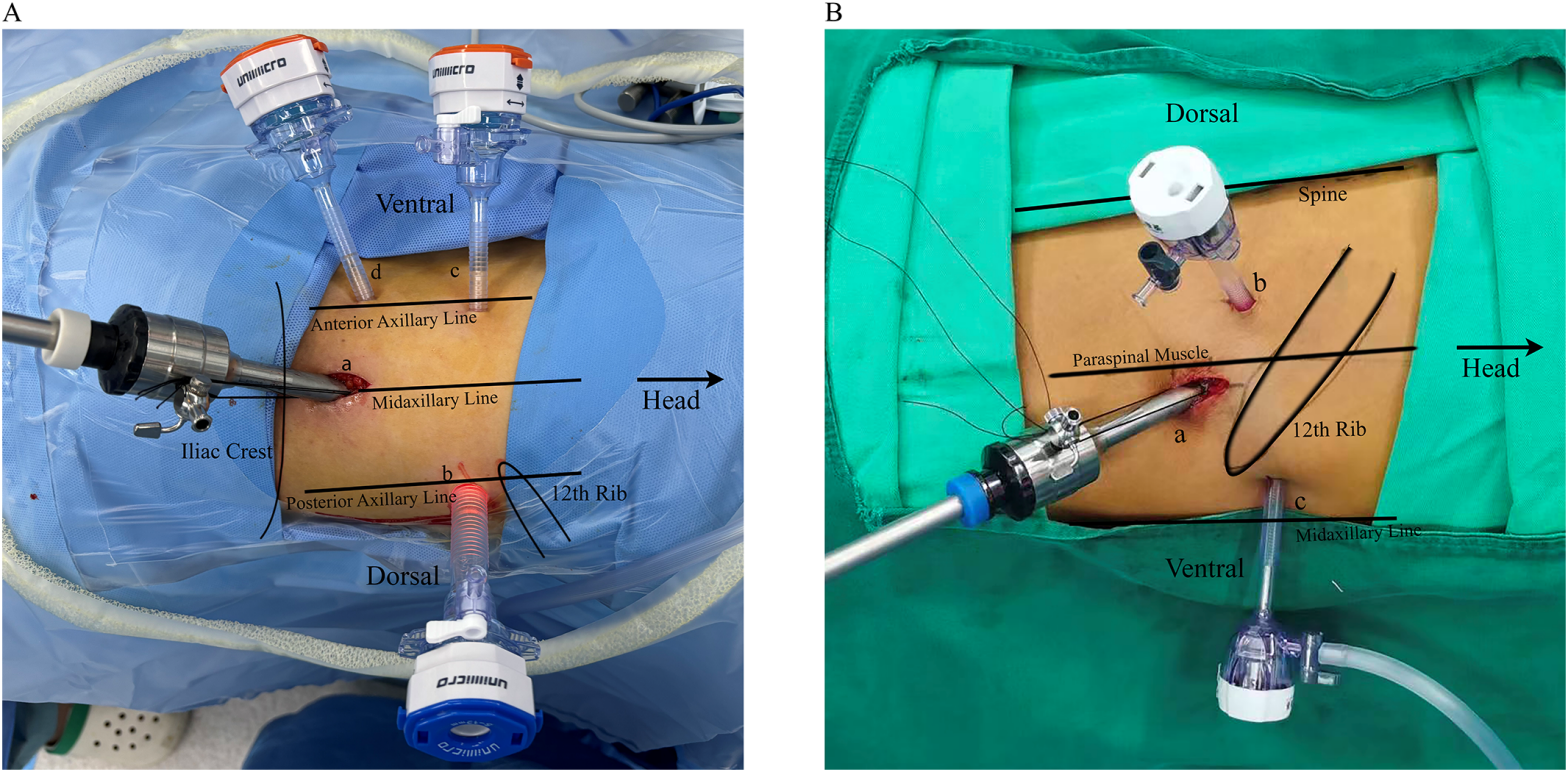
**(A)** Left-sided lateral retroperitoneal approach (LRA) routinely utilizing four trocars: (a) 10-mm camera trocar, (b) 12-mm trocar, (c) 5-mm trocar, and (d) 5-mm assistant trocar. **(B)** Right-sided posterior retroperitoneal approach (PRA) utilizing three trocars: (a) 10-mm camera trocar, (b) 5-mm trocar, and (c) 5-mm trocar.

**Figure 2:**
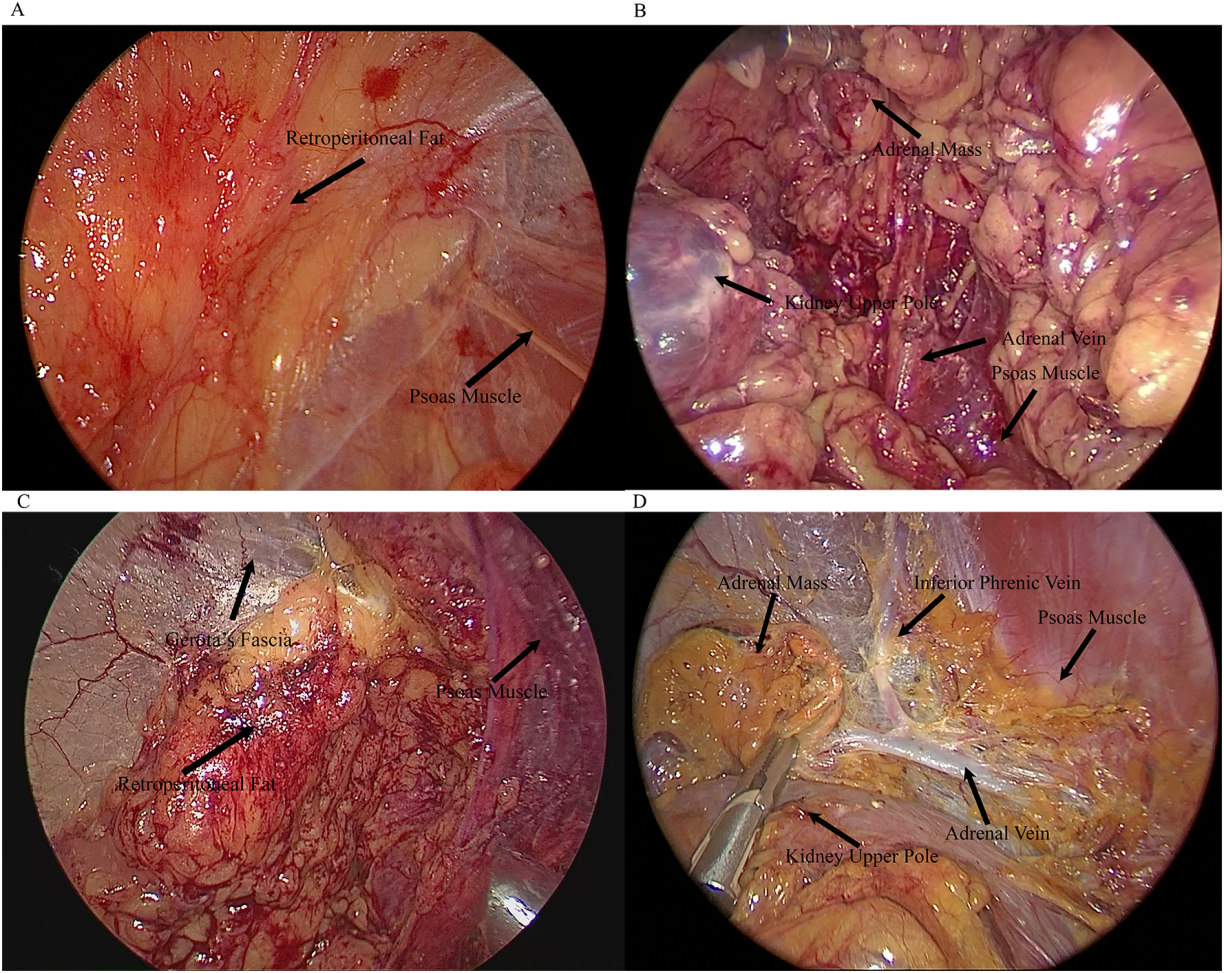
Intraoperative views. **(A)** Entry into the retroperitoneal space via lateral retroperitoneal approach (LRA). **(B)** Dissection of the adrenal mass and vein via LRA. **(C)** Entry into the retroperitoneal space via posterior retroperitoneal approach (PRA). **(D)** Dissection of the adrenal mass and vein via PRA.

Patients were placed in the full flank position on the operating table; the affected side was then hyper extended by elevating the lumbar bridge of the table. A 4-trocar technique was used routinely as shown in **Figure 1A**. The first incision was made between the bottom of the ribcage and the iliac crest in the midaxillary line which is 1.5cm in length as the camera port (**Figure 1A, a**, 10mm trocar),. The retroperitoneal space was bluntly dissected by a finger and afterwards a custom-made balloon inflated with 500–700 mL of air. The second trocar was inserted in the posterior axillary line and below the 12th rib (**Figure 1A, b**, 12mm for left-side and 5mm for right-side tumor). The third trocar was installed above the iliac crest at anterior axillary line (**Figure 1A, c**, 5mm for left-side and 12mm for right-side tumor). Under laparoscopic guidance, the fourth was inserted under the subcostal margin at the anterior axillary line as assistant port (**Figure 1A, d**, 5mm). Following carbon dioxide insufflation to establish the retroperitoneal working space, the retroperitoneal adipose tissue was dissected using an ultrasonic scalpel (Harmonic Scalpel, Ethicon, Johnson & Johnson). Subsequently, Gerota’s fascia was incised and the renal upper pole mobilized to achieve adrenal gland exposure. The operative fields when entering the retroperitoneal space (**Figure 2A**) and after dissecting the adrenal gland (**Figure 2B**) were shown in **Figure 2**. Then the adrenal vein was ligated early to avoid the risk of excessive catecholamine secretion and tumor disruption (13). For the right adrenalectomy, the inferomedial aspect of the gland was carefully separated from the renal vein and inferior vena cava, while the lateral and superior surfaces were dissected. During the left adrenalectomy, dissection the left renal artery helped in identifying adrenal vein (14). After the ligation of adrenal vein and inferior phrenic vessels, the superior surface was dissected, followed by the dissection of lateral and inferomedial surface. The adrenal gland was placed in an endo-bag, and removed in 12mm Trocar. The drain tube was inserted into the assistant port.

#### 2.2.2 PRA

Post-anesthesia, the patient was positioned jack-knifed, and an initial incision was made in 1.5cm length below the tip of the 12th rib along the border of the paraspinal muscle for a camera port (**Figure 1B, a**, 10mm). The retroperitoneal space was bluntly dissected using a finger, followed by inflation of a custom-made balloon with 500–700 mL of air. Two more ports were added over 4cm medially (**Figure 1B, b**, 5mm) and laterally (**Figure 1B, c**, lower tip of the 11th rib, 5mm) to initial cut’s position. After the preparation of the trocars, the operative procedures were performed similarly to LRA. The operative fields when entering the retroperitoneal space (**Figure 2C**) and after dissecting the adrenal gland (**Figure 2D**) were shown in **Figure 2**. For patients with less intraoperative adipose tissue dissection and minimal bleeding, no drainage tube was left postoperatively.

### 2.3 Statistical analysis

All data were analyzed using R software, version 4.3.1. Continuous variables were compared using unpaired or paired two-tailed Student’s t-tests, Kruskal-Wallis tests, paired Wilcoxon tests, and categorical variables were compared using χ^2^-tests and Fisher’s exact tests. “MatchIt” package was used to develop propensity score matching (PSM) to balance the potential confounding variables between LRA and PRA groups. After excluding the bilateral cases, the propensity score included preoperative characteristics (sex, age, tumor laterality, BMI, tumor size, hypertension and diabetes mellitus status) and matched with a 1:1 ratio. Differences were considered statistically significant at p<0.05. Univariate and multivariate logistic regression models were utilized with “glm()” function of “broom” package, and factors with p<0.10 in univariate analysis underwent stepwise multivariate analysis, with statistical significance set at p<0.05. “gtsummray” package was complemented to obtain the odds ratio (OR) and 95% confidence interval (CI) of logistic regression analyses.

## 3 Results

### 3.1 Baseline demographics

Clinicopathologic characteristics for the overall cohort and subgroups are summarized in **Table 1**. Generally, the patients’ average age was 53.8 ± 13.0 years with mean BMI of 24.8 ± 3.27 kg/m^2^, among which 93(50.3%) patients were male. All patients were Chinese Han population. According to the included patients’ medical history, 123(66.5%) had hypertension while 29(15.7%) suffered diabetes mellitus. ASA scores were distributed as follows: grade I (n=39, 21.1%), II (n=143, 77.3%), and III (n=3, 1.6%). Tumor laterality was left-sided (n=101, 54.6%), right-sided (n=81, 43.8%), or bilateral (n=3, 1.6%; all managed via PRA). Median tumor diameter measured 2.3 cm (2.80 ± 1.19 cm, range: 1.0–6.5 cm), with 27 (14.6%) tumors ≥4 cm. According to the surgical techniques, 90 (48.6%) patients underwent PRA while the rest (95, 51.4%) took LRA. Pathological classifications comprised functional tumors (Conn’s syndrome [n=28, 15.1%], pheochromocytoma [n=24, 13.0%], Cushing’s syndrome [n=2, 1.1%]) and non-functional lesions (benign adenoma [n=129, 69.7%], metastasis [n=2, 1.1%]). Baseline characteristics showed no significant differences between LRA and PRA subgroups. After 1:1 PSM that excluded bilateral cases, the BMI of LRA patients (24.3 ± 3.11) was found to be significantly lower than that of PRA patients (25.0 ± 3.14; *p* = 0.013). Importantly, no significant differences were observed in other baseline characteristics between groups after propensity score matching.

**Table 1:** Baseline characteristics of the patients.

Characteristics	Overall (n=185)	Before PSM			After PSM		
		LRA (n=95)	PRA (n=90)	p	LRA (n=87)	PRA (n=87)	p
Age (year)	53.8 ± 13.0	53.6 ± 13.6	53.9 ± 12.4	0.876^∗^	54.0 ± 13.5	53.9 ± 12.4	0.953^**^
Sex (%)							
Male	93 (50.3)	48 (50.5)	45 (50.0)		41 (47.1)	43 (49.4)	
Female	92 (49.7)	47 (49.5)	45 (50.0)	0.940^†^	46 (52.9)	44 (50.6)	0.879^†^
Hypertension (%)	123 (66.5)	62 (65.3)	61 (67.8)	0.837^†^	55 (63.1)	59 (67.8)	0.632^†^
Diabetes mellitus (%)	29 (15.7)	14 (14.7)	15 (16.7)	0.874^†^	14 (16.1)	14 (16.1)	1^†^
BMI (kg/m3)	24.8 ± 3.27	25.2 ± 3.39	24.4 ± 3.09	0.089^∗^	25.0 ± 3.11	24.3 ± 3.14	0.013^**^
ASA score (%)							
I	39 (21.1)	24 (25.3)	15 (16.7)		23 (26.4)	14 (16.1)	
II	143 (77.3)	69 (72.6)	74 (82.2)		63 (72.4)	72 (82.7)	
III	3 (1.6)	2 (2.1)	1 (1.1)	0.151^‡^	1 (1.2)	1 (1.2)	0.201^‡^
Tumor laterality (%)							
Right	81 (43.8)	41 (43.2)	40 (44.4)		37 (42.5)	40 (45.8)	
Left	101 (54.6)	54 (56.8)	47 (52.2)		50 (57.5)	47 (54.2)	
Bilateral	3 (1.6)	0 (0)	3 (3.3)	0.332^‡^	0 (0)	0 (0)	0.760^†^
Tumor size (cm)	2.80 ± 1.19	2.83 ± 1.25	2.70 ± 1.11	0.432^∗^	2.82 ± 1.20	2.69 ± 1.13	0.438^**^
Size<4cm (%)	158 (85.4)	80 (84.2)	78 (86.7)		74 (85.1)	75 (86.2)	
Size≥4cm (%)	27 (14.6)	15 (15.8)	12 (13.3)	0.791^†^	13 (14.9)	12 (13.8)	1^†^
Preoperative pathology (%)							
Functional tumors							
Conn’s syndrome	28 (15.1)	17 (17.9)	11 (12.2)		13 (14.9)	11 (12.6)	
Pheochromocytoma	24 (13.0)	13 (13.7)	11 (12.2)		12 (13.8)	11 (12.6)	
Cushing’s syndrome	2 (1.1)	0 (0)	2 (2.2)		0 (0)	2 (2.3)	
Adrenocortical carcinoma	0 (0)	0 (0)	0 (0)		0 (0)	0 (0)	
Nonfunctional tumors							
Benign adenoma	129 (69.7)	64 (67.3)	65 (72.3)		61 (70.1)	62 (71.3)	
Metastasis	2 (1.1)	1 (1.1)	1 (1.1)	0.577^‡^	1 (1.2)	1 (1.2)	0.836^‡^

^∗^Two-tailed t-test. **Paired two-tailed t-test. † χ2 test. ‡ Fisher’s exact test. PSM, propensity score match; SD, standard deviation; LRA, lateral retroperitoneoscopic adrenalectomy; PRA, posterior retroperitoneoscopic adrenalectomy; BMI, body mass index; ASA, American Society of Anesthesiologist.

### 3.2 Comparison of perioperative characteristics between LRA and PRA subgroups

Perioperative metrics are detailed in **Table 2**. Mean operative time was significantly shorter for PRA (56.2 ± 13.7 minutes; range: 30–100 minutes) compared to LRA (79.0 ± 22.8 minutes; range: 40–145 minutes; *p* < 0.001). Hemoglobin change (1.26 ± 0.41 g/dL) did not differ significantly between groups (*p* = 0.06), and no conversions to open surgery occurred. Intraoperative complications included blood pressure fluctuations (n=18, 9.7%) and transfusions (n=6, 3.2%). Postoperatively, 28 (15.1%) patients developed complications. Clavien-Dindo grade I–II was defined minor, including fever (12, 6.5%), hypokalemia (7, 3.8%), pneumonia (4, 2.2%) and arrhythmia (1, 0.5%), while Clavien-Dindo grade III–V was defined major (surgical ICU admission, 4, 2.2%). Complication rates were comparable between groups (*p* > 0.05). Median hospital stay was shorter for PRA (4 days) versus LRA (6 days; *p* < 0.001). The indwelling rate of drainage tubes and time to drain removal were significantly higher in the LRA group (95, 100%; 3.98 ± 1.50 days) than in the PRA group (38, 42.2%; 3.42 ± 1.29 days). After PSM, Wilcoxon test for paired samples showed no significance of retention time of the drainage tubes between LRA and PRA groups.

**Table 2:** Perioperative characteristics of the patients.

Perioperative characteristics	Overall (n=185)	Before PSM			After PSM		
		LRA (n=95)	PRA (n=90)	p	LRA (n=87)	PRA (n=87)	p
Operative time (minutes)							
Unilateral	68.1 ± 22.1	79.0 ± 22.8	56.2 ± 13.7	<0.001^∗^	79.2 ± 22.9	56.2 ± 13.7	<0.001^**^
Bilateral	–	–	179, 120 and 110	–	–	–	–
Preoperative hemoglobin (g/dL)	13.0 ± 0.96	13.0 ± 0.97	13.1 ± 0.94	0.245^∗^	13.0 ± 0.99	13.1 ± 0.94	0.178^**^
Postoperative hemoglobin (g/dL)	11.8 ± 1.05	11.6 ± 1.10	11.9 ± 0.99	0.071^∗^	11.6 ± 1.12	11.9 ± 0.99	0.053^**^
Change in hemoglobin (g/dL)	1.26 ± 0.41	1.32 ± 0.50	1.20 ± 0.28	0.060^∗^	1.33 ± 0.51	1.21 ± 0.28	0.055^**^
Intraoperative complications (%)							
Open conversion	0 (0)	0 (0)	0 (0)	1^†^	0 (0)	0 (0)	1^†^
Hemodynamic instability	18 (9.7)	10 (10.5)	8 (8.9)	0.89^†^	7 (8.0)	8 (9.2)	1^†^
Intraoperative blood transfusion	6 (3.2)	5 (5.3)	1 (1.1)	0.212^‡^	3 (3.4)	1 (1.2)	0.621^‡^
Postoperative complications (%)							
Minor							
Fever	12 (6.5)	6 (6.3)	6 (6.7)	0.923^†^	5 (5.7)	6 (6.9)	1^†^
Hypokalemia	7 (3.8)	1 (1.1)	6 (6.7)	0.059^‡^	1 (1.2)	5 (5.7)	0.211^‡^
Pneumonia	4 (2.2)	4 (4.2)	0 (0)	0.143^‡^	3 (3.4)	0 (0)	0.246^‡^
Arrhythmia	1 (0.5)	1 (1.1)	0 (0)	1^‡^	1 (1.2)	0 (0)	1^‡^
Major							
Surgical ICU admission	4 (2.2)	4 (4.2)	0 (0)	0.143^‡^	3 (3.4)	0 (0)	0.246^‡^
Postoperative hospitalization (days, mean ± SD (range))	5.22 ± 1.81 (2-13)	5.91 ± 1.79 (3-13)	4.48 ± 1.50 (2-10)	<0.001^#^	5.86 ± 1.73 (3-13)	4.41 ± 1.47 (2-10)	<0.001^&^
Drain	133 (71.9)	95 (100)	38 (42.2)	<0.001^†^	87 (100)	36 (41.4)	<0.001^†^
Time to drain removal (days, mean ± SD (range))		3.98 ± 1.50 (1-9)	3.42 ± 1.29 (2-8)	0.014^#^	4.00 ± 1.51 (1-9)	3.36 ± 1.29 (2-8)	0.441^&^

*Two-tailed t-test. **Paired two-tailed t-test. † χ^2^ test. ^‡^ Fisher’s exact test. # Kruskal-Wallis test. & Wilcoxon test for paired samples. PSM, propensity score match; SD, standard deviation; LRA, lateral retroperitoneoscopic adrenalectomy; PRA, posterior retroperitoneoscopic adrenalectomy; ICU, intensive care unit.

### 3.3 Regression analyses of operative time and hospital stay

#### 3.3.1 Operative duration stratification

Patients were stratified into ≤60-minute (n=92, 49.7%) and >60-minute (n=93, 50.3%) cohorts (**Table 3**). Univariate logistic regression analysis identified BMI (OR = 1.17, 95% CI: 1.07-1.29, *p* = 0.001), male sex (OR = 2.35, 95% CI: 1.31-4.28, *p* = 0.004) and LRA approach (PRA reference, OR = 0.18, 95% CI: 0.09-0.33, *p* < 0.001) as predictors of prolonged surgery. Multivariate analysis confirmed these factors (BMI: OR = 1.14, 95% CI: 1.03-1.27, *p* = 0.017; male sex: OR = 2.47, 95% CI: 1.26-4.93, *p* = 0.009; LRA: OR = 0.17, 95% CI: 0.08-0.32, *p* < 0.001) as independent variables.

**Table 3:** Perioperative clinical factors associated with operative time of >60 minutes for RA and postoperative hospital days of ≥ 5 days after RA.

Factors	Univariate analysis			Multivariate analysis			Univariate analysis			Multivariate analysis		
	OR	95% CI	p	OR	95% CI	p	OR	95% CI	p	OR	95% CI	p
Age	1.00	0.98-1.02	0.985	–	–	–	1.01	0.98-1.03	0.649	–	–	–
BMI	1.17	1.07-1.29	0.001^†^	1.14	1.03-1.27	0.017^∗^	0.97	0.89-1.06	0.500	–	–	–
Sex												
Female	reference	–	–	reference	–	–	reference	–	–	–	–	–
Male	2.35	1.31-4.28	0.004^†^	2.47	1.26-4.93	0.009^∗^	0.81	0.45-1.46	0.491	–	–	–
Pathology												
Non-functional	reference	–	–	–	–	–	reference	–	–	reference	–	–
Functional	1.55	0.82-2.95	0.181	–	–	–	2.20	1.12-4.47	0.025^†^	2.26	1.08-4.91	0.034*
Tumor size	1.06	0.82-1.38	0.665	–	–	–	1.05	0.81-1.38	0.670	–	–	–
Tumor laterality												
Left	reference	–	–	–	–	–	reference	–	–	–	–	–
Right	1.04	0.58-1.86	0.907	–	–	–	0.72	0.39-1.30	0.273	–	–	–
Bilateral	NA	NA	0.985	–	–	–	NA	NA	0.986	–	–	–
ASA												
I	reference	–	–	–	–	–	reference	–	–	–	–	–
II	1.50	0.74-3.11	0.269	–	–	–	0.99	0.48-2.02	0.979	–	–	–
III	NA	NA	0.985	–	–	–	NA	NA	0.986	–	–	–
Surgical technique												
LRA	reference	–	–	reference	–	–	reference	–	–	reference	–	–
PRA	0.18	0.09-0.33	<0.001^†^	0.17	0.08-0.32	<0.001^∗^	0.26	0.14-0.48	<0.001^†^	0.40	0.17-0.91	0.030^∗^
Hypertension												
No	reference	–	–	–	–	–	reference	–	–	–	–	–
Yes	1.19	0.65-2.21	0.568	–	–	–	1.09	0.58-2.03	0.784	–	–	–
Diabetes mellitus												
No	reference	–	–	–	–	–	reference	–	–	–	–	–
Yes	0.79	0.35-1.75	0.566	–	–	–	0.69	0.31-1.54	0.357	–	–	–
Operative time				–			1.03	1.02-1.05	<0.001^†^	1.02	1.01-1.04	0.012^∗^
Change in hemoglobin							1.01	0.94-1.09	0.719	–	–	–
Drain												
No							reference	–	–	–	–	–
Yes							2.67	1.39-5.19	0.003^†^	1.43	0.62-3.31	0.404
Intraoperative complications												
No				–			reference	–	–	–	–	–
Yes				–			3.02	1.05-10.9	0.057^†^	2.35	0.25-2.81	0.731
Postoperative complications												
No				–			reference	–	–	reference	–	–
Minor				–			3.19	0.98-14.3	0.078^†^	2.58	0.14-1.67	0.251
Major				–			1.62	0.19-13.8	0.634	1.46	0.02-6.63	0.323

RA, retroperitoneoscopic adrenalectomy; CI, confidence interval; OR, odds ratio; BMI, body mass index; ASA, American Society of Anesthesiologist physical status classification; LRA, lateral retroperitoneoscopic adrenalectomy; PRA, posterior retroperitoneoscopic adrenalectomy. † p<0.10 and involve in multivariate analysis. ∗p<0.05.

#### 3.3.2 Hospital stay analysis

Post-stay duration was categorized as <5 days (n=75, 40.5%) or ≥5 days (n=110, 59.5%) based on the median value (**Table 3**). Univariate analysis associated prolonged stays with operative time (OR = 1.03 per minute, CI:1.02–1.05, *p* < 0.001), functional tumors (non-functional reference, OR = 2.20, CI:1.12–4.47, *p* = 0.025), PRA approach (LRA reference, OR = 0.26; CI:0.14–0.48; *p* < 0.001), intraoperative complications (trend: OR = 3.02, CI:1.05–10.9, *p* = 0.057), drainage retention (OR = 2.67, CI:1.39-5.19, *p* = 0.003) and minor postoperative events (trend: OR = 3.19, CI:0.98–14.3, *p* = 0.078). Multivariate analysis retained functional tumors (non-functional reference, OR = 2.26, CI:1.08–4.91, *p* = 0.034), operative time (OR = 1.02 per minute; CI:1.01–1.04, *p* = 0.012) and PRA approach (LRA reference, OR = 0.40; CI:0.17–0.91, *p* = 0.030) as independent predictors.

## 4 Discussion

Surgeons consistently prioritize minimally invasive techniques that optimize safety, efficacy, and postoperative recovery. Given the adrenal gland’s deep anatomical location (15–18), multiple approaches have been developed for adrenalectomy. While lateral transabdominal adrenalectomy (LTA) remains a gold standard (19), retroperitoneal adrenalectomy (RA)—encompassing both posterior (PRA) and lateral (LRA) approaches—has emerged as a viable alternative (20). A recent network meta-analysis highlighted PRA/LRA superiority in minimizing morbidity and operative duration among minimally invasive techniques (21). Our study, the largest comparative analysis to date (n=185), demonstrates that PRA significantly reduces both operative time (*p* < 0.001) and postoperative hospitalization (*p* < 0.001) versus LRA while maintaining comparable intraoperative blood loss, conversion rates (none observed), and perioperative complication profiles—validating its safety-efficacy balance as reported previously (10, 11).

LRA’s popularity stems from familiarity with lateral retroperitoneal anatomy—a mainstay in renal surgery. This approach offers expanded working space and visualization advantages for larger tumors (>4–5 cm) or suspected malignancies (20). However, the LRA approach necessitates a more intricate navigation through more anatomical structures to access the adrenal glands, extending the operative time. Meanwhile, based on our medical center’s experience, abdominal fat shifts ventrally under gravity in the prone position. Concurrently, since trocars are placed closer to the dorsal side, after balloon dilation of the retroperitoneal field, fat is further pushed ventrally. This results in less adipose tissue overlying Gerota’s fascia compared to LRA, reducing time spent on clearing retroperitoneal fat and minimizing intraoperative bleeding. Additionally, given that trocar placement is anatomically closer to the adrenal gland and central adrenal vein, once surgeons become familiar with anatomical variations in the prone position, dissection around the adrenal gland and central vein can be achieved more rapidly compared to lateral positioning. During the initial phase of surgical practice, postoperative drainage volume following PRA was observed to be minimal. Thus, for patients undergoing uneventful procedures with limited intraoperative blood loss, drain placement was intentionally omitted. This approach reduced operative duration without increasing postoperative complications, while facilitating earlier patient discharge. PRA also allows bilateral adrenal tumor operations without patient repositioning, aiding recovery (22). Yet, PRA’s posterior approach may be unfamiliar to surgeons who have to convert from transperitoneal to retroperitoneal procedures, its confined working space implies a steeper learning curve, and it may be unsuitable for larger tumors or malignancies (23). Also, PRA has difficulty quickly converting to open surgery during major bleeding (14). In present study, all surgeons involved in performing PRA procedures had already fully mastered the LRA technique prior to implementation and possessed comprehensive understanding of retroperitoneal anatomical structures. This foundational knowledge reserve may have effectively facilitated accelerated transition between surgical methods, thereby ensuring enhanced operational safety during clinical application of PRA.

Our study found that PRA had significantly shorter operative times and postoperative hospital stays compared to LRA, with comparable blood loss. Although PRA’s perioperative complications seemed lower than LRA’s, the difference wasn’t statistically significant. BMI, sex, and surgical methods were identified as independent factors affecting RA operative times over 60 minutes. As was observed in other studies, it took longer time to perform operations on patients with higher BMI due to more fatty tissues, which makes it difficult to maintain sufficient working space. Intriguingly, operation took longer in males, which has also been reported in previous studies (10, 24, 25), and other retroperitoneal surgeries, like partial nephrectomy (26). The likely reasons are the presence of more muscles, deeper location of the tumors and more adherent perinephric and retroperitoneal fatty tissues (24). Compared with LRA, the PRA technique dramatically reduced the operative time, which is consistent with the results of previous studies. Oh et al. reported the operative time for PRA was reduced by 33.9 minutes compared to LRA (71.5 ± 31.5 vs 105.4 ± 41.2 minutes, PRA vs LRA) (10), while Agha et al.’s research demonstrated that PRA had a 42-minute shorter surgical duration than LRA (80 ± 43 vs 122 ± 25 minutes, PRA vs LRA) (11). However, the size of tumors was not related to surgical time, probably due to the few cases of large tumors (tumors ≥ 4cm, n=27, 14.6%; tumors ≥ 6cm, n=4, 2.2%) in present study. Longer operative times, functional tumors, LRA approach, retention of drainages and complication occurrences lengthened recovery time. However, the multivariate analysis revealed that only functional tumors, operative time and surgical methods were independent indicators. Meanwhile, we have identified significant variations in the length of hospital stays between different countries. A study from Austria revealed that the average postoperative hospital stay for PRA patients was 3.63 days, which is comparable with our findings, while LTA patients had a longer hospitalization period of 8.84 days (7). A German study indicated that the mean hospital stays for partial adrenalectomy and total adrenalectomy in RA (retroperitoneoscopic adrenalectomy) cases were 3.2 days and 3.7 days, respectively, with the maximum recorded duration reaching 15 days (27). A study from Yale School of Medicine revealed that 86% of patients can be discharged within two days after surgery (28). As a result, we need to refine medical strategies to shorten postoperative hospital stays for patients while ensuring their safety and reducing healthcare expenditures. Overall, PRA appears to be a more efficient method, reducing operative time and hospital stay without compromising patient safety.

Our study included three patients with bilateral adrenal neoplasms, all of whom underwent PRA with operative times of 179, 120, and 110 minutes. The identified pathologies of the tumors were benign adenoma, with the largest measuring 3.0 cm. All three patients were discharged from the hospital within a week without perioperative complications. These results suggest PRA’s effectiveness and safety for treating bilateral adrenal lesions. However, further studies are needed due to the small number of cases. In our study, four patients who underwent LRA were admitted to ICU post-operation. The diagnosed tumor pathologies in these cases were all pheochromocytoma and the tumor sizes were 2.0cm, 3.1cm, 3.5cm and 4.0cm in diameter. The primary reason for ICU admission was hemodynamic instability, and all these patients were transferred back to the urology ward the day after surgery. Notably, none of the patients experienced severe surgical complications such as open conversion or death. This suggests that RA approaches are generally safe when performed by experienced surgeons.

Our retrospective single-center design inherently limits generalizability. Additional constraints include: (1) As a retrospective study, patients were not randomly assigned to LRA or PRA groups. The surgical approach was primarily determined based on the surgeons’ comprehensive assessment and clinical experience. (2)Underrepresentation of large tumors/malignancies precluding robust assessment of RA’s efficacy in complex cases; (3) Surgeon experience variability potentially confounding outcomes; (4) Absence of comparisons with LTA or robotic approaches—a critical direction for future multicenter trials.

## 5 Conclusions

In conclusion, both lateral and posterior retroperitoneoscopic adrenalectomy (LRA and PRA) demonstrate comparable safety and efficacy for adrenal tumor resection, with similar perioperative complication rates and blood loss. However, PRA offers distinct advantages, including reduced operative duration and shorter postoperative hospitalization. Prolonged operative times were associated with elevated BMI, male sex, and the LRA approach. Further studies involving larger adrenal masses are warranted to validate the generalizability of these findings and refine patient selection criteria.

## Data Availability

The raw data supporting the conclusions of this article will be made available by the authors, without undue reservation.
